# Assessment of neonatologists' competency in managing gestational diabetes complications: a cross-sectional analysis from China

**DOI:** 10.3389/fendo.2025.1574480

**Published:** 2025-07-03

**Authors:** Yi Yang, Yao Yang

**Affiliations:** Department of Neonatal, The Central Hospital of Enshi Tujia and Miao Autonomous, Enshi, Hubei, China

**Keywords:** gestational diabetes mellitus, prevalence, neonatology, KAP, China cardiomyopathy, alongside significantly

## Abstract

**Background:**

Gestational diabetes mellitus (GDM) significantly impacts long-term child health outcomes. This study assessed neonatologists' knowledge, attitudes, and practices (KAP) regarding GDM-related complications in offspring.

**Methods:**

A cross-sectional study of 1,614 neonatologists in Hubei Province, China, utilized a validated 28-item questionnaire examining knowledge (12 items), attitudes (8 items), and practices (8 items). Responses were scored on a trichotomous scale. Binary logistic regression analyzed predictors of satisfactory performance across domains.

**Results:**

Among 1,614 neonatologists, 1,437 (89%) demonstrated satisfactory knowledge, 1,513 (94%) positive attitudes, and 1,165 (72%) good practices. Knowledgeable practitioners were significantly older (45.4 vs 36.2 years; OR 1.42 [95% CI 1.40-1.44]; *p*<.001) with greater experience (13.8 vs 10.5 years; 1.41 [1.35-1.46]; *p*<.001). Academic hospital affiliation showed higher competency versus community settings (0.12 [0.08-0.20]; *p*<.001). Practice patterns varied by education, with MD-PhD holders demonstrating higher odds of good practice (1.32 [1.03-1.71]; P=.032) compared with fellowship training (0.69 [0.51-0.92]; *p*=.009). Universal documentation of maternal GDM coexisted with suboptimal rates of periodic evaluations (81%) and specialist referrals (84%). Knowledge competency (7.52 [5.90-9.60]; *p*<.001) and positive attitudes (15.81 [9.90-25.26]; *p*<.001) strongly predicted practice patterns.

**Conclusions:**

Despite high knowledge levels and positive attitudes, particularly among experienced practitioners in academic settings, implementation gaps exist in follow-up protocols and specialist referrals. Practice setting significantly influences care delivery, suggesting the need for standardized protocols across healthcare tiers.

## 1 Introduction

Gestational diabetes mellitus (GDM) is defined as glucose intolerance first recognized during pregnancy and has emerged as one of the most prevalent metabolic complications in obstetric care (1, 2). Studies indicates that *in utero* exposure to maternal hyperglycemia predisposes children to long‐term metabolic disturbances—including obesity, insulin resistance, and cardiovascular risk factors—that may persist into adulthood (3, 4). Such adverse sequelae underscore the critical importance of early recognition and proactive management strategies to mitigate future morbidity in these high‐risk populations (5). According to a meta-analysis, women with a history of GDM are at a substantially elevated risk of developing T2DM and cardiovascular diseases in their subsequent years. A comprehensive meta-analysis encompassing 20 studies with a total of 1,332,373 participants (67,956 women with prior GDM and 1,264,417 controls) revealed that those with a GDM history have an almost 10-fold increased likelihood of progressing to T2DM compared to women who maintained normoglycemia during pregnancy (6).

Neonatologists directly influence long-term health outcomes by identifying clinical manifestations and stratifying risk in infants of mothers with GDM (7, 8). Effective clinical management requires deep understanding of the pathophysiologic cascade from intrauterine diabetic exposure to childhood metabolic dysfunction (3, 9). Current evidence demonstrates clear links between GDM and childhood complications, yet data on neonatologists' knowledge, attitudes, and practices (KAP) regarding these outcomes remains limited (8, 10).

In the immediate post-partum period, infants born to mothers with GDM exhibit a distinctive constellation of morbidities that extend well beyond the classic triad of macrosomia, hypoglycemia, and shoulder dystocia. Contemporary meta-analyses document 3- to 10-fold increases in symptomatic neonatal hypoglycemia, respiratory-distress syndrome, and hypertrophic cardiomyopathy, alongside significantly higher incidences of polycythemia, disordered iron homeostasis, and transient myocardial dysfunction (11, 12). Large multicenter cohorts further confirm that GDM-exposed newborns have an adverse composite outcome rate exceeding 50%, compared with <20% in normoglycemic pregnancies (13, 14).

In China, the National Health Commission’s 2023 Specification for the Management of High-Risk Newborns mandates that tertiary-level neonatal departments operate structured follow-up clinics for 24 to 36 months post-discharge, especially for infants with intrauterine exposure to maternal hyperglycaemia (15–17). These clinics encompass scheduled assessments of growth trajectories, neurodevelopment, and early metabolic derangements (18). Similarly, the American Academy of Pediatrics Clinical Practice Guideline for Infants of Diabetic Mothers recommends that neonatologists initiate cardiometabolic surveillance, endocrine referrals, and interdisciplinary care planning beginning in the immediate postnatal period (16, 19). These frameworks underscore a global shift in neonatology from acute stabilization to longitudinal risk mitigation. As such, neonatologists serve not only as first-line responders at birth but also as gatekeepers of long-term metabolic and developmental health in GDM-exposed offspring. Assessing their knowledge, attitudes, and practices (KAP) regarding long-term complications is therefore essential for identifying implementation gaps within this extended continuum of care.

China has witnessed a marked escalation in gestational diabetes mellitus (GDM) prevalence over the past two decades, with pooled national rates nearing 15% (20–22). creating a significant inter-generational cardiometabolic burden for both mothers and offspring. Concurrently, healthcare directives, including National Health Commission regulations and American Academy of Pediatrics guidance, now mandate extended follow-up for GDM-exposed infants by neonatologists, typically through 24–36 months of age. This pivotal role allows neonatologists to intervene during the early-childhood “window of developmental plasticity,” addressing latent metabolic derangements (23–25). This signifies a crucial evolution in neonatal practice: moving beyond the stabilization of acute complications to proactively anticipating, stratifying, and mitigating long-term risks. Given this pressing epidemiological context, shifting regulatory expectations, and considerable regional disparities in resource allocation, our study investigates the KAP of neonatologists in Hubei Province concerning these long-term GDM-related complications. By identifying current strengths and implementation deficits, we seek to inform targeted educational strategies, standardized clinical protocols, and policy adjustments aimed at disrupting the inter-generational cycle of metabolic disease.

## 2 Methodology

### 2.1 Study design and setting

This cross-sectional study was conducted to assess neonatologists’ KAP regarding long-term complications in children born to mothers with GDM. The study was conducted across multiple healthcare institutions in Hubei Province, China, from June 2021 to October 2024. The study followed the Strengthening the Reporting of Observational Studies in Epidemiology (STROBE) guidelines (26, 27).

### 2.2 Questionnaire

A 28-item KAP instrument—originally developed for obstetric and primary-care clinicians managing GDM—was adapted for use with Chinese neonatologists. The English source tool was forward-translated into simplified Chinese by two independent bilingual experts, reconciled, and back-translated to ensure semantic equivalence. A five-member expert panel (two neonatologists, one pediatric endocrinologist, one epidemiologist, one medical-education specialist) assessed relevance and clarity, yielding an item-content-validity index (I-CVI) of 0.94 and a scale-level CVI/Ave of 0.92. Pilot testing with 40 neonatologists (excluded from the main analysis) produced Cronbach’s α = 0.88 and a two-week test–retest intraclass-correlation coefficient of 0.91—values comparable to those reported in prior Chinese KAP studies among obstetric nurses and community physicians (α 0.85–0.91) (10, 28–37). The final questionnaire retained the parent tool’s three-domain architecture (knowledge = 12 items; attitudes = 8 items; practices = 8 items) and trichotomous response format (“Yes,” “Maybe,” “No”).

#### 2.2.1 Distribution strategy

To maximize reach across diverse clinical settings, the survey link was disseminated through hospital e-mail networks, provincial neonatology-association listservs, and WeChat professional groups. QR codes linked to the online questionnaire were posted in neonatal-unit staff lounges and displayed at regional neonatology conferences. Participants had three weeks to respond; two reminder e-mails (one week apart) were issued. At academic and tertiary centers, designated survey coordinators facilitated distribution and follow-up. For community and private hospitals, direct outreach via professional forums and peer champions was employed to enhance uptake.

#### 2.2.2 Response-rate optimization

The multi-channel approach, combined with periodic reminders and on-site QR promotion, achieved a final sample of 1,614 eligible neonatologists—well above the minimum calculated requirement and yielding > 80 % statistical power for planned subgroup analyses.

### 2.3 Participation inclusion and sample size

Study participation required board-certified neonatologists practicing in Hubei Province healthcare institutions, with a minimum of 2 years of direct clinical experience and documented management of GDM-exposed neonates. All participants provided informed consent. Exclusion criteria encompassed incomplete questionnaire responses, lack of GDM case exposure, and those in administrative positions without direct patient care within the past 2 years. Neonatologists on extended leave (>6 months) or working exclusively in research without clinical duties were also excluded. The stringent selection criteria ensured data quality and relevant clinical expertise for comprehensive analysis.

Sample size determination incorporated the pooled GDM prevalence of 14.8% (95% CI, 12.8%-16.7%) from a meta-analysis of 79,064 Chinese participants (27). The minimum sample size was calculated with Cochran’s formula $n_{0}=\frac{Z_{1-\alpha /2}^{2}p\;(1-p)}{d^{2}}$ . where Z=1.96 (for 95% confidence), *p*=0.148(estimated prevalence), and *d*=0.025. This yielded a minimum sample size of *n*_0_=776. To account for potential design effects and non-response, the sample was inflated by 20%, resulting in a target of 931 participants. Ultimately, 1,614 eligible neonatologists completed the questionnaire, exceeding the minimum requirement and providing >80% power for subgroup analyses.

### 2.4 Measurements

This cross-sectional study utilized a validated questionnaire examining knowledge, attitudes, and practices regarding GDM complications. The 28-item instrument assessed three domains: knowledge of metabolic, cardiovascular, and neurodevelopmental sequelae (12 items); attitudes toward screening and interdisciplinary management (8 items); and implementation of evidence-based practices (8 items). Response options followed a trichotomous scale (Yes/No/Maybe). Knowledge assessment scored "Yes" as 1 point, "Maybe" as 0.5 points, and "No" as 0 points, with a maximum achievable score of 12. Attitude evaluation assigned 2 points for "Yes", 1 point for "Maybe", and 0 for "No", yielding a maximum score of 16. Practice assessment followed identical scoring criteria as attitudes, with a maximum of 16 points. Domain competency thresholds were established at 80% of maximum scores (≥9.6 for knowledge, ≥12.8 for attitudes and practices), defining knowledgeable status, positive attitudes, and good practice patterns. All domains underwent standardized categorization for statistical analysis, with scores above thresholds classified as satisfactory performance. In addition to the dichotomous competency cut-points, we calculated mean ± SD, median (IQR), and full range for each KAP domain; these descriptive metrics are presented in **Supplementary Table S1**.

### 2.5 Statistical analysis

Statistical analyses were conducted using R version 4.3.2 (R Foundation for Statistical Computing). Continuous variables were reported as mean ± standard deviation (SD), and categorical variables as frequencies and percentages. Between-group comparisons were performed using the Wilcoxon rank-sum test for continuous variables and Pearson’s Chi-squared test for categorical variables.

Binary logistic regression models were constructed to identify predictors of satisfactory knowledge and practice scores, with outcome variables dichotomized as satisfactory versus unsatisfactory. Covariates were selected *a priori* based on established frameworks and prior literature related to KAP assessments in diabetes care. These included age, gender, years of clinical experience, highest academic qualification (MD, MD-PhD, or fellowship), and practice setting (academic, tertiary, community, or private hospital).

Variables with a *p*-value < 0.20 in univariate analysis were retained for multivariable modelling to avoid premature exclusion of relevant predictors. Additionally, variables recognized in previous research as potential confounders or effect modifiers were included. Multicollinearity was assessed using variance inflation factor (VIF), and all retained variables had VIF < 2. Model fit was evaluated using the Hosmer–Lemeshow test, and explanatory power was assessed using Nagelkerke’s R². Adjusted odds ratios (aORs) with 95% confidence intervals (CIs) were reported, and statistical significance was defined as *p* < 0.05.

### 2.6 Ethics statement

Ethical approval was obtained from the Department of Neonatal, The Central Hospital of Enshi Prefecture Tujia and Miao Autonomous Prefecture (EA:2021/24/R3/145). All procedures conformed to the Declaration of Helsinki. Participation was voluntary; an electronic informed-consent statement was displayed on the first page of the survey, and completion of the questionnaire constituted consent. No personal identifiers were collected.

## 3 Results

Of 1,614 participating neonatologists, mean age was 44 (SD 7) years with 13·4 (SD 5·3) years of clinical experience. The cohort comprised 944 (58%) males and 670 (42%) females. Educational qualifications included MD (844 [52%]), MD-PhD (485 [30%]), and neonatology fellowship (285 [18%]). Practice settings were distributed across academic hospitals (473 [29%]), tertiary care centers (476 [29%]), community hospitals (341 [21%]), and private practices (324 [20%]). Assessment outcomes revealed 1,437 (89%) participants as knowledgeable, 1,513 (94%) with positive attitudes, and 1,165 (72%) demonstrating good practice patterns regarding GDM-related complications, as shown in **Table 1**. However, the mean scores were 10.5 ± 1.4 for knowledge (median 11; IQR 10–12), 14.2 ± 1.8 for attitudes (median 15; IQR 13–16), and 12.9 ± 2.1 for practices (median 13; IQR 12–15), with respective ranges of 4–12, 6–16, and 5–16 (**Supplementary Table S1**). These values align with competency thresholds (≥9.6 for knowledge; ≥12.8 for attitudes and practices), indicating generally high performance, though greater variability was observed in practice scores (**Supplementary Table S1**).

**Table 1:** Baseline demographic and professional characteristics (N = 1,614) of the participants, including age, gender, education, clinical experience, practice setting, and distributions of knowledge, practice, and attitude status.

Variable	N = 1,614^1^
Age	44 ± 7
Gender	
Female	670 (42%)
Male	944 (58%)
Education	
MD	844 (52%)
MD-PhD	485 (30%)
Neonatology Fellowship	285 (18%)
Experience	13.4 ± 5.3
Practice Setting	
Academic Hospital	473 (29%)
Community Hospital	341 (21%)
Private Practice	324 (20%)
Tertiary Care Center	476 (29%)
Knowledge	
Knowledgeable	1,437 (89%)
Not-Knowledgeable	177 (11%)
Practice	
Bad Practice	449 (28%)
Good Practice	1,165 (72%)
Attitude	
Negative Attitude	101 (6.3%)
Positive Attitude	1,513 (94%)

^1^Mean ± SD; n (%).

Assessment of knowledge domains revealed universal recognition (100%) of GDM's association with type 2 diabetes risk, metabolic outcomes, and the importance of maternal glycemic control. High awareness (>90%) was demonstrated for insulin resistance (1,556 [96%]), neurodevelopmental impacts (1,534 [95%]), and renal complications (1,504 [93%]). Lower recognition was observed for lipid abnormalities (1,341 [83%]) and epigenetic influences (1,393 [86%]), as shown in **Table 2**.

**Table 2:** Distribution of responses (“Maybe,” “No,” “Yes”) to key knowledge statements regarding GDM-related metabolic and neonatal complications.

Knowledge assessment	Response		
	Maybe	No	Yes
Statement			
Evidence supports GDM-induced insulin resistance.	58 (3.6%)	0 (0%)	1,556 (96%)
Fetal hyperinsulinemia drives metabolic dysregulation.	64 (4.0%)	0 (0%)	1,550 (96%)
GDM-induced epigenetic changes affect child health.	191 (12%)	30 (1.9%)	1,393 (86%)
GDM contributes to neonatal lipid abnormalities.	154 (9.5%)	119 (7.4%)	1,341 (83%)
GDM is associated with renal complications.	70 (4.3%)	40 (2.5%)	1,504 (93%)
GDM is linked to type 2 diabetes risk.	0 (0%)	0 (0%)	1,614 (100%)
GDM is tied to early cardiovascular issues.	151 (9.4%)	0 (0%)	1,463 (91%)
GDM negatively affects neurodevelopment.	80 (5.0%)	0 (0%)	1,534 (95%)
Genetics and intrauterine factors interact in GDM.	128 (7.9%)	0 (0%)	1,486 (92%)
Intrauterine hyperglycemia predisposes neonates to obesity.	54 (3.3%)	58 (3.6%)	1,502 (93%)
Maternal GDM increases offspring metabolic risk.	0 (0%)	0 (0%)	1,614 (100%)
Maternal glycemic control impacts neonatal outcomes.	0 (0%)	0 (0%)	1,614 (100%)
Total	950 (4.9%)	247 (1.3%)	18,171 (94%)

Attitude assessment showed unanimous agreement (1,614 [100%]) on GDM as a key risk factor and the value of proactive care. Strong support was evident for additional training (1,574 [98%]), guideline adequacy (1,584 [98%]), and early metabolic screening (1,559 [97%]). Interdisciplinary care received relatively lower, though still substantial, support (1,502 [93%]), as shown in **Table 3**.

**Table 3:** Summary of neonatologists’ responses to attitude statements on training, guidelines, risk identification, and interdisciplinary care for GDM management.

Attitude assessment	Response		
	Maybe	No	Yes
Statement			
Additional training is needed.	40 (2.5%)	0 (0%)	1,574 (98%)
Current guidelines are adequate.	30 (1.9%)	0 (0%)	1,584 (98%)
Early metabolic screening is essential.	55 (3.4%)	0 (0%)	1,559 (97%)
Early risk identification is vital.	50 (3.1%)	115 (7.1%)	1,449 (90%)
GDM should be a key risk factor.	0 (0%)	0 (0%)	1,614 (100%)
Interdisciplinary care is critical.	58 (3.6%)	54 (3.3%)	1,502 (93%)
More research will improve outcomes.	84 (5.2%)	64 (4.0%)	1,466 (91%)
Proactive care reduces complications.	0 (0%)	0 (0%)	1,614 (100%)
Total	317 (2.5%)	233 (1.8%)	12,362 (96%)

Practice patterns demonstrated universal documentation of maternal GDM (1,614 [100%]) and high adherence to metabolic screening (1,550 [96%]). However, periodic evaluations (1,314 [81%]) and endocrinology referrals (1,358 [84%]) showed lower implementation rates. Evidence-based guideline adherence was reported by 1,459 (90%) participants. All domain comparisons showed significant differences (*p*<0·001), as shown in **Table 4**.

**Table 4:** Frequencies of “Maybe,” “No,” and “Yes” responses assessing adherence to evidence-based practices in the management of GDM and related neonatal care.

Practice assessment	Response		
	Maybe	No	Yes
Statement			
I adhere to evidence-based guidelines.	98 (6.1%)	57 (3.5%)	1,459 (90%)
I apply to interdisciplinary management.	76 (4.7%)	96 (5.9%)	1,442 (89%)
I conduct metabolic screening consistently	0 (0%)	64 (4.0%)	1,550 (96%)
I document maternal GDM in records.	0 (0%)	0 (0%)	1,614 (100%)
I follow a standard follow-up protocol.	145 (9.0%)	98 (6.1%)	1,371 (85%)
I perform cardiovascular assessments.	186 (12%)	50 (3.1%)	1,378 (85%)
I refer at-risk neonates to endocrinologists.	87 (5.4%)	169 (10%)	1,358 (84%)
I schedule periodic evaluations.	94 (5.8%)	206 (13%)	1,314 (81%)
Total	686 (5.3%)	740 (5.7%)	11,486 (89%)

In multivariate analysis of knowledge competency (**Table 5**), knowledgeable practitioners were significantly older (45·4 vs 36·2 years; OR 1·42 [95% CI 1·40-1·44]; *p*<0·001) and predominantly male (61·9% vs 30·5%; 3·71 [2·64-5·19]; *p*<0·001). Experience emerged as a strong predictor (13·8 vs 10·5 years; 1·41 [1·35-1·46]; *p*<0·001). Practice setting significantly influenced knowledge levels (*p*<0·001), with academic hospitals showing higher competency compared to community hospitals (0·12 [0·08-0·20]; *p*<0·001) and private practices (0·30 [0·18-0·52]; *p*<0·001). Good practice patterns (772% vs 311%; 752 [536-1060]; *p*<0·001) and positive attitudes (970% vs 672%; 11 [063-158]; *p*<0·001) were significantly associated with knowledge competency.

**Table 5:** Binary logistic regression analysis identifying demographic and professional predictors of being “Knowledgeable” versus “Not-Knowledgeable” about GDM complications.

Variable	Knowledgeable	Not-knowledgeable	*p*-value^2^	Binary logistic regression		
	(N = 1,437)^1^	(N = 177)		Coefficient (B)	OR (95% CI)^3^	*p*-value
**Age**	45.4 ± 7.0	36.2 ± 2.9	<0.001	0.351	1.42 (1.40, 1.44)	<0.001
Gender			<0.001	1.309	3.71 (2.64, 5.19)	<0.001
Female	547 (38.1%)	123 (69.5%)				
Male	890 (61.9%)	54 (30.5%)				
Education			0.2	–	Overall: 0.20	0.2
MD	743 (51.7%)	101 (57.1%)		*Ref*		
MD-PhD	432 (30.1%)	53 (29.9%)		0.104	1.11 (0.78, 1.58)	0.57
Neonatology Fellowship	262 (18.2%)	23 (13.0%)		0.438	1.55 (0.96, 2.49)	0.07
Experience	13.8 ± 5.1	10.5 ± 5.5	<0.001	0.34	1.41 (1.35, 1.46)	<0.001
Practice Setting			<0.001	–	Overall: <0.001	<0.001
Academic Hospital	452 (31.5%)	21 (11.9%)		*Ref*		
Community Hospital	248 (17.3%)	93 (52.5%)		–2.091	0.12 (0.08, 0.20)	<0.001
Private Practice	281 (19.6%)	43 (24.3%)		–1.192	0.30 (0.18, 0.52)	<0.001
Tertiary Care Center	456 (31.7%)	20 (11.3%)		0.057	1.06 (0.57, 1.98)	0.85
Practice Status			<0.001	2.019	7.52 (5.36, 10.60)	<0.001
Bad Practice	327 (22.8%)	122 (68.9%)				
Good Practice	1,110 (77.2%)	55 (31.1%)				
Attitude Status			<0.001	0.131	1.1 (0.63, 1.58)	<0.001
Negative Attitude	43 (3.0%)	58 (32.8%)				
Positive Attitude	1,394 (97.0%)	119 (67.2%)				

*^1^* Mean ± SD; n (%); *^2^* Wilcoxon rank sum test; Pearson’s Chi-squared test; *^3^* Binary logistic Regression.

Attitude analysis (**Table 6**) revealed that positive attitudes were associated with increased age (44·6 vs 41·6 years; OR 1·28; *p*=0·002) and male gender (59·5% vs 43·6%; 1·90 [1·27-2·85]; *p*=0·002). MD-PhD holders demonstrated lower odds of positive attitudes compared to MDs (0·19 [0·12-0·30]; *p*<0·001). Knowledge competency strongly predicted positive attitudes (15·81 [9·90-25·26]; *p*<0·001), showed good practice patterns (7·11 [4·78-10·57]; *p*<0·001).

**Table 6:** Logistic regression analysis examining factors associated with a positive versus negative attitude towards GDM management among neonatologists.

Variable	Negative attitude	Positive attitude	*p*-value^2^	Binary logistic regression		
	N = 58^1^	N = 1,556^1^		Coefficient (B)	OR (95% CI)^3^	*p*-value
Age	41.6 ± 4.7	44.6 ± 7.4	0.002	0.25 (–)	1.28	0.002
Gender			0.002	0.64 (0.21)	1.90 (1.27 – 2.85)	0.002
Female	57 (56.4%)	613 (40.5%)				
Male	44 (43.6%)	900 (59.5%)				
Education			<0.001			
MD	24 (23.8%)	820 (54.2%)		*Ref*		
MD-PhD	65 (64.4%)	420 (27.8%)		–1.67 (0.25)	0.19 (0.12 – 0.30)	<0.001
Neonatology Fellowship	12 (11.9%)	273 (18.0%)		–0.41 (0.36)	0.67 (0.36 – 1.26)	0.26
Experience	16.2 ± 4.1	13.3 ± 5.3	<0.001	–0.35 (–)	0.71	<0.001
Practice Setting			0.001			
Academic Hospital	22 (21.8%)	451 (29.8%)		*Ref*		
Community Hospital	36 (35.6%)	305 (20.2%)		–0.88 (0.28)	0.41 (0.26 – 0.62)	0.0016
Private Practice	22 (21.8%)	302 (20.0%)		–0.40 (0.31)	0.67 (0.38 – 1.19)	0.2
Tertiary Care Center	21 (20.8%)	455 (30.1%)		0.06 (0.31)	1.06 (0.60 – 1.87)	0.86
Knowledge			<0.001	2.76 (0.22)	15.81 (9.90 – 25.26)	<0.001
Knowledgeable	43 (42.6%)	1,394 (92.1%)				
Not-Knowledgeable	58 (57.4%)	119 (7.9%)				
Practice			<0.001	1.96 (0.23)	7.11 (4.78 – 10.57)	<0.001
Bad Practice	71 (70.3%)	378 (25.0%)				
Good Practice	30 (29.7%)	1,135 (75.0%)				

^1^Mean ± SD; n (%);^2^Wilcoxon rank sum test; Pearson’s Chi-squared test; ^3^Binary logistic Regression.

Practice pattern assessment (**Table 7**) showed that good practice was associated with increased age (46·0 vs 40·2 years; OR 1·32 per year; *p*<0·001) and varied by education level, with MD-PhD holders showing higher odds (1·32 [1·03-1·71]; *p*=0·032) compared to fellowship-trained practitioners (0·69 [0·51-0·92]; *p*=0·009). Practice setting significantly influenced outcomes (*p*<0·001), with private practices showing higher odds of good practice (3·42 [1·31-4·22]; *p*<0·001) compared to community hospitals (0·35 [0·02-0·93]; *p*<0·001). Knowledge competency (7·52 [5·90-9·60]; *p*<0·001) and positive attitudes (0·14 [0·09-0·22]; *p*<0·001) were strongly associated with practice patterns.

**Table 7:** Regression analysis determining predictors of good versus bad clinical practice in managing long-term complications related to maternal GDM.

Variable	Bad practice	Good practice	*p*-value^2^	Binary logistic regression		
	N = 449^1^	N = 1,165^1^		Coefficient (B)	OR (95% CI)^3^	*p*-value
Age	40.2 ± 5.5	46.0 ± 7.2	<0.001	0.28 (–)	1.32 (per year)	<0.001
Gender			0.3	–0.12 (0.11)	0.89 (0.72 – 1.10)	0.3
Female	177 (39.4%)	493 (42.3%)				
Male	272 (60.6%)	672 (57.7%)				
Education			<0.001			
MD	236 (52.6%)	608 (52.2%)		*Ref*		
MD-PhD	110 (24.5%)	375 (32.2%)		0.28 (0.13)	1.32 (1.03 – 1.71)	0.032
Neonatology Fellowship	103 (22.9%)	182 (15.6%)		–0.38 (0.15)	0.69 (0.51 – 0.92)	0.009
Experience	13.4 ± 7.0	13.4 ± 4.5	0.2	0.00 (–)	1.0 (0.01 - 0.33)	0.2
Practice Setting			<0.001			
Academic Hospital	119 (26.5%)	354 (30.4%)		*Ref*		
Community Hospital	168 (37.4%)	173 (14.8%)		–1.06 (0.15)	0.35 (0.02 - 0.93)	<0.001
Private Practice	29 (6.5%)	295 (25.3%)		1.23 (0.22)	3.42 (1.31 - 4.22)	<0.001
Tertiary Care Center	133 (29.6%)	343 (29.4%)		–0.14 (0.15)	0.87 (0.55 - 1.35)	0.35
Knowledge			<0.001	2.02 (0.17)	7.52 (5.90 – 9.60)	<0.001
Knowledgeable	327 (72.8%)	1,110 (95.3%)				
Not-Knowledgeable	122 (27.2%)	55 (4.7%)				
Attitude			<0.001	–1.96 (0.23)	0.14 (0.09 – 0.22)	<0.001
Negative Attitude	71 (15.8%)	30 (2.6%)				
Positive Attitude	378 (84.2%)	1,135 (97.4%)				

^1^Mean ± SD; n (%);^2^Wilcoxon rank sum test; Pearson’s Chi-squared test; ^3^Binary logistic Regression.

**Figure 1:**
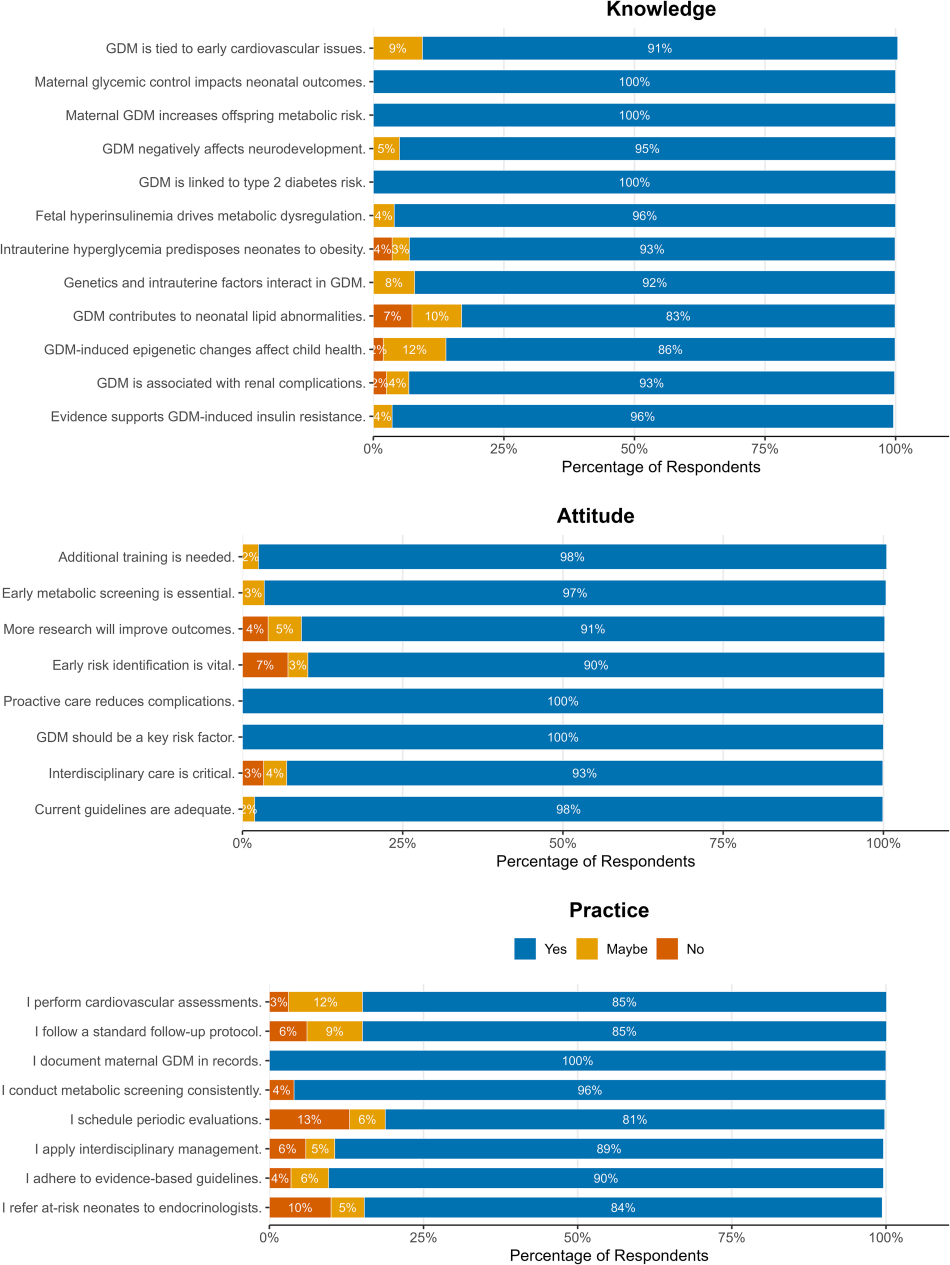
Distribution of responses to all 28 items of knowledge, attitude, and practice (KAP) questionnaire administered to neonatologists (N = 1,614) regarding GDM-related neonatal and childhood complications. The knowledge domain (top) demonstrates overall high awareness, with lower recognition of lipid abnormalities and epigenetic mechanisms. The attitude and practice domains (middle and bottom) reflect strong endorsement of screening and interdisciplinary care but highlight notable deficits in routine follow-up scheduling, endocrinology referrals, and adherence to standardized postnatal protocols. All questionnaire items and response frequencies are fully represented in this figure.

Knowledge domain showed universal recognition of core GDM outcomes, with lower awareness of epigenetic (86%) and lipid effects (83%). Attitudes revealed complete agreement on risk assessment and proactive care, while practice patterns demonstrated optimal maternal documentation but suboptimal implementation of follow-up (81%) and specialist referrals (84%) (**Figure 1**).

## 4 Discussion

The study of neonatologists' knowledge, attitudes, and practices concerning long-term complications in children born to mothers with GDM reveals significant insights into the understanding and management of this prevalent condition. The findings indicate a high level of awareness among the participating neonatologists regarding the association of GDM with various health risks in offspring, which aligns with existing literature. However, discrepancies in knowledge and practice patterns highlight areas for improvement and further research. The high proportion of satisfactory knowledge in our cohort appears to arise from an interplay of mandatory training, case-mix exposure, and structured professional development rather than academic degree alone. Neonatology board certification in China requires rotations through obstetric medicine and perinatal endocrinology, guaranteeing baseline familiarity with maternal metabolic disorders, while routine care of infants of diabetic mothers provides continual experiential learning. Since 2022, national regulations have also obliged neonatologists to complete at least 15 hours of continuing medical education (CME) annually, including dedicated modules on neonatal metabolic disease and GDM-related sequelae; senior clinicians—who receive greater CME funding and protected study leave—therefore accrue more guideline familiarity than early-career staff (38–40). Comparable, training-linked gains in GDM knowledge have been documented among midwives, obstetric nurses, and multidisciplinary after-care teams following structured educational programs (10, 29).

The universal recognition of GDM's association with type 2 diabetes risk and metabolic outcomes among the neonatologists is particularly noteworthy. This aligns with findings from Landon et al., who emphasized the long-term health implications of maternal diabetes on offspring, including increased risks for obesity and metabolic syndrome (41). Furthermore, the high awareness (>90%) regarding insulin resistance and neurodevelopmental impacts corroborates the conclusions of Adane et al., who conducted a systematic review highlighting the cognitive development challenges faced by children born to mothers with diabetes (42). The recognition of renal complications (93%) also reflects the established understanding of the multifaceted risks associated with GDM, as noted by Hammoud et al., who discussed the long-term BMI and growth profiles in offspring of women with gestational diabetes (43).

In China, tertiary neonatal departments are required by the National Health Commission’s 2023 *Specification for the Management of High-Risk Newborns* to operate structured follow-up clinics for up to 36 months, with explicit protocols for growth, metabolic, and neurodevelopmental surveillance of infants exposed to maternal hyperglycemia (17, 44). Within this framework, neonatologists initiate early endocrine referral, implement dietary and physical-activity counselling for caregivers, and coordinate data transfer to community pediatric services. Our finding that 89 % of neonatologists possessed satisfactory knowledge yet only 72 % reported guideline-concordant practice indicates a critical implementation gap at the very point in the care continuum where long-term risk can be intercepted. Targeted continuing-medical-education modules standardized electronic follow-up templates, and stronger referral links with pediatric endocrinology could bridge this knowledge-practice divide and help realize the intent of current national policy (45–47).

Few studies have focused specifically on neonatologists’ KAP regarding GDM. Most existing surveys have examined obstetricians, midwives, primary-care physicians, or pediatricians. A national survey of Chinese obstetricians reported satisfactory knowledge in 78 % but adherence to postpartum screening protocols in only 62 % of respondents; similar knowledge–practice discordances have been documented among obstetric nurses in Egypt (31) and antenatal clinicians in Australia (30). By contrast, our cohort demonstrated higher knowledge competency (89 %) yet displayed a practice adherence rate (72%) that mirrors the implementation shortfalls seen in these other professional groups (28, 29, 48). Collectively, these findings suggest that the obstacle is less a deficiency of awareness and more the absence of streamlined, resource-appropriate pathways to translate knowledge into consistent clinical action—an issue that appears to transcend specific provider roles and healthcare systems.

However, the study revealed lower recognition rates for lipid abnormalities (83%) and epigenetic influences (86%). This discrepancy may be attributed to the evolving nature of research in these areas. For instance, while the connection between GDM and lipid metabolism is acknowledged, it may not be as widely emphasized in clinical training compared to more established associations such as obesity and diabetes (49). The emerging field of epigenetics, particularly concerning maternal health and fetal development, is still gaining traction in clinical discussions, which could explain the relatively lower awareness among practitioners (50).

The assessment of attitudes among neonatologists regarding GDM reveals a strong consensus on the importance of proactive care and the necessity for additional training. The unanimous agreement (100%) on GDM being a key risk factor is consistent with the literature, which emphasizes the critical role of early identification and management of GDM to mitigate long-term complications for both mothers and their offspring (51, 52). The high support for additional training (98%) and guideline adequacy (98%) reflects a recognition of the evolving nature of diabetes management and the need for continuous professional development, which is echoed in studies that highlight the positive impact of educational interventions on healthcare providers' knowledge and practices (53, 54).

Moreover, early post-discharge screening refers to routine metabolic and neuro-developmental surveillance of GDM-exposed infants—including serial glucose checks, lipid profiling, growth-trajectory plotting, and age-appropriate developmental assessments—carried out during the first 24-36 months of life (55, 56). This proactive approach is vital, as early detection can significantly reduce the incidence of complications associated with GDM, including obesity and metabolic disorders in offspring (57, 58). The relatively lower support for interdisciplinary care (93%) suggests an area for potential growth, as collaborative approaches have been shown to enhance patient outcomes in diabetes management (58–60). The literature indicates that interdisciplinary teams can provide comprehensive care that addresses the multifaceted needs of patients with GDM, thereby improving adherence to treatment protocols and health outcomes (49, 60–63).

The finding that 72% of neonatologists demonstrated good practice patterns regarding GDM-related complications raises questions about the remaining 28% who did not. This gap in practice may reflect systemic issues, such as inadequate training or resources, which have been noted in other studies examining healthcare providers' responses to maternal diabetes (64–66). For instance, while Morgan et al. (67) found that healthcare professionals often recognize the risks associated with maternal diabetes, they may lack the tools or protocols necessary to implement effective management strategies. Moreover, the positive attitudes reported by the neonatologists could be influenced by recent guidelines and educational initiatives aimed at improving care for mothers with GDM. The literature suggests that continuous professional development and updated clinical guidelines can significantly enhance healthcare providers' confidence and practices regarding GDM management (68, 69). However, the persistence of knowledge gaps, particularly regarding lipid abnormalities and epigenetic factors, indicates a need for targeted educational interventions to address these specific areas (70, 71).

Questionnaire findings highlight operational deficiencies and knowledge gaps likely contributing to suboptimal patient management. Firstly, long-term follow-up was suboptimal: only 81% of respondents scheduled periodic evaluations beyond the newborn phase, and 84% consistently referred high-risk infants to paediatric endocrinology. Qualitatively-identified key barriers included limited subspecialist availability, absent national follow-up templates, and high patient-to-clinician ratios. Secondly, knowledge lacunae, especially regarding lipid abnormalities (83% correct) and epigenetic mechanisms (86% correct), may reduce perceived urgency for prolonged surveillance of these less-familiar sequelae, potentially reinforcing care discontinuities. Addressing these intertwined systemic and knowledge challenges—via unified electronic follow-up pathways, tele-endocrinology support, and targeted CME on less-recognized long-term complications—is crucial for bridging the knowledge-practice gap.

The association between good practice patterns and positive attitudes, as well as knowledge competency, reinforces the interconnectedness of these domains. This finding is supported by previous research that indicates a strong correlation between healthcare providers' knowledge, attitudes, and their subsequent practices (72–75). The positive attitudes associated with increased age and male gender may reflect generational differences in training and exposure to GDM management, as well as potential biases in the healthcare workforce (76, 77). Interestingly, MD-PhD holders demonstrating lower odds of positive attitudes compared to MDs suggests that the dual focus on research and clinical practice may impact their engagement with clinical guidelines and patient care (78, 79). Although 90 % of respondents affirmed adherence to evidence-based protocols, national chart-audit studies from tertiary neonatal units’ report documented follow-up rates closer to 70%. This gap underscores classic social-desirability bias inherent to questionnaire surveys and reinforces our recommendation for future linkage studies using electronic medical records or prospective audit-and-feedback designs.

This study represents the first comprehensive investigation of neonatologists' KAP regarding GDM complications in China, with robust methodology including stratified sampling and validated assessment tools. The large sample size (N=1,614) and diverse practice settings enhance generalizability within Chinese healthcare. However, several limitations warrant consideration. The cross-sectional design and self-reported data preclude causal inference and may introduce response bias. Geographic restriction to Hubei Province, potential digital access barriers, and lack of longitudinal assessment limit broader insights. Because data were self-reported, results may overestimate true adherence; future studies should incorporate chart audits or direct observation. Additionally, the absence of patient outcome correlation and practice verification through medical records restrict clinical impact evaluation. These limitations suggest opportunities for future prospective, multi-provincial studies incorporating objective practice assessment and outcome measures.

## 5 Conclusion

Our findings demonstrate strong knowledge foundation and positive attitudes among neonatologists regarding GDM-related complications, particularly in academic settings and among experienced practitioners. The significant association between practice settings and care delivery patterns, with private practices showing superior implementation compared to community hospitals, highlights systemic variations in care standards. While knowledge levels are high across domains, the notable gaps in follow-up care and specialist referrals suggest organizational barriers to optimal practice implementation. These results emphasize the need for standardized protocols across healthcare tiers and enhanced support for community hospitals to ensure consistent care delivery for GDM-exposed neonates.

## Data Availability

The raw data supporting the conclusions of this article will be made available by the authors, without undue reservation.
