# Supplementary material for: Assessment of neonatologists' competency in managing gestational diabetes complications: a cross-sectional analysis from China
Source: Front Endocrinol (Lausanne). 2025 Jul 3;16:1574480. doi: 10.3389/fendo.2025.1574480 (PMC12268886; doi:10.3389/fendo.2025.1574480)
Supplement: Supplementary file 1 [file Table1.docx]

**Table S1. KAP Domain Score Distribution Among Neonatologists (N = 1,614)**

| **Domain** | **Maximum score** | **Mean ± SD** | **Median (IQR)** | **Range** |
| --- | --- | --- | --- | --- |
| Knowledge | 12 | 10.5 ± 1.4 | 11 (10–12) | 4–12 |
| Attitudes | 16 | 14.2 ± 1.8 | 15 (13–16) | 6–16 |
| Practices | 16 | 12.9 ± 2.1 | 13 (12–15) | 5–16 |
| Raw scores for knowledge (max = 12), attitudes (max = 16), and practices (max = 16) domains are presented as mean ± standard deviation (SD), median (interquartile range, IQR), and full range. Competency was defined as achieving ≥80% of the maximum score per domain (i.e., ≥9.6 for knowledge; ≥12.8 for attitudes and practices). | | | | |
